# The Effects of Lavender and Citrus aurantium on Anxiety and Agitation of the Conscious Patients in Intensive Care Units: A Parallel Randomized Placebo-Controlled Trial

**DOI:** 10.1155/2021/5565956

**Published:** 2021-06-15

**Authors:** Zahra Karimzadeh, Mansooreh Azizzadeh Forouzi, Elham Rahiminezhad, Mehdi Ahmadinejad, Mahlagha Dehghan

**Affiliations:** ^1^Student Research Committee, Razi Faculty of Nursing and Midwifery, Kerman University of Medical Sciences, Kerman, Iran; ^2^Neuroscience Research Center, Institute of Neuropharmacology, Kerman University of Medical Sciences, Kerman, Iran; ^3^Fellow of Critical Care Medicine, Kerman University of Medical Sciences, Kerman, Iran; ^4^Nursing Research Center, Department of Critical Care Nursing, Razi Faculty of Nursing and Midwifery, Kerman University of Medical Sciences, Kerman, Iran

## Abstract

**Background:**

Conscious patients admitted to intensive care units (ICU) suffer from anxiety and agitation for various reasons, which can affect their recovery processes.

**Aims:**

To compare the effects of lavender and Citrus aurantium essential oils on anxiety and agitation of conscious patients admitted to ICUs.

**Design:**

A randomized parallel placebo-controlled trial.

**Methods:**

One hundred and fifty conscious patients admitted to ICUs were selected by convenience sampling and were randomly divided into three groups, groups of lavender aromatherapy and Citrus aurantium aromatherapy, in addition to the routine care and inhalation of five drops of lavender or Citrus aurantium essential oils for 30 minutes. The placebo group, in addition to routine care, was provided with 5 drops of normal saline for 30 minutes. Anxiety was assessed with the state subscale of State-Trait Anxiety Inventory, and agitation was examined with Richmond Agitation-Sedation Scale before, immediately, one hour, and three hours after the intervention.

**Results:**

All three groups suffered from relatively severe state anxiety before the intervention. The level of anxiety in the lavender and Citrus aurantium groups was significantly lower than that of the placebo group immediately and three hours after the intervention (*P* < 0.05). No significant difference was observed between the two groups of lavender and Citrus aurantium. The majority of the samples in all three groups were agitated before the intervention, but agitation of all three groups decreased after the intervention. Restless/agitation reduced significantly in all three groups. Although restless/agitation of the lavender and Citrus aurantium groups reduced more than that of the placebo, no significant difference was found between the three groups.

**Conclusion:**

The results of the present study showed the positive effects of lavender aromatherapy and Citrus aurantium aromatherapy on reducing the anxiety of patients admitted to ICUs. *Relevance to Clinical Practice*. Aromatherapy can be used as an effective and safe intervention to reduce anxiety in ICUs.

## 1. Introduction

Patients admitted to intensive care units (ICU), especially conscious patients, are under a lot of stress and experience varying degrees of anxiety and agitation from internal and external sources [1]. The experiences of conscious patients admitted to the ICU show that some measures cause fear, anxiety, and pain in patients, including the presence of several patients with different conditions in a single room or the admission of a newly ill patient, resuscitation of patients, visits of different doctors, some nursing procedures, the presence of various equipment, crowded ward, no observance of the patient's privacy, and patient's sleep disorders [2]. Some conscious patients believe that inattention of nurses, improper communication, and inattention to the needs of conscious patients have led to patient-nurse distrust and thus anxiety and agitation of patients [1, 2].

Anxiety is an emotional state characterized by feelings of tension, anger, worry, fear, and increased activity of the autonomic system, leads to psychophysical response, changes vital signs, and increases the rate of mortality in ICUs [3, 4]. In a study in Jordan, 84.3% of ICU patients experience anxiety [3]. Anxiety changes vital signs, increases the need for oxygen, and then influences patient's cardiac output and hemodynamic status. Increased anxiety leads to increased blood pressure, respiratory rate, tachycardia, and agitation of the patient [5].

Agitation is a state of anxiety or nervous excitement that is accompanied by sudden and intense movements and unpredictable behaviors due to discomfort [6, 7]. Agitation occurs in critically ill patients frequently with varying degrees accompanied with severe and persistent tremors, bed disorganization, and removal of the endotracheal tube and IV lines [4, 6, 8]. Agitated ICU patients face problems such as prolonged stay in intensive care units, removal of arterial and venous catheters, increased oxygen consumption, failure to participate in therapeutic interventions, injury to staff and patient, excessive use of sedatives, high costs, complications, and mortality [4, 6, 8].

In general, there are two pharmacological and nonpharmacological treatments for anxiety and agitation [6]. Pharmacological treatment often includes side effects of drops in blood pressure, impaired vital functions such as respiration and heart rate, drowsiness, nausea and vomiting, and allergic reactions and shock; numerous physical and psychological complications; and drug dependence and imposes high costs on the healthcare system [9]. Therefore, today, there is a growing tendency to use nonpharmacological methods and complementary medicine such as aromatherapy [10].

Aromatherapy is a type of complementary medicines in which oils and aromas are used to prevent and treat diseases [10–12]. Lavender is a plant, in which essential oil is used for aromatherapy. This plant affects heart function, stimulates blood circulation, and is effective in creating peace of mind [13]. Lavender has antidepressant, antispasmodic, and antibacterial properties and is known to be effective in treating insomnia, anxiety, and pain [12, 14]. In traditional medicine, Citrus aurantium flowers are used to treat neurological diseases such as hysteria and weakness of the nerves. This plant is also known as a sedative, soporific, stomachic, and relief of heart palpation [15].

Studies have investigated the effects of lavender aromatherapy and Citrus aurantium aromatherapy on anxiety and agitation in different patients, but limited studies have investigated the effects of these two aromas on anxiety and agitation of conscious patients in ICUs. Studies show different results about the effectiveness of aromatherapy. Karadag et al. studied the effect of lavender on the anxiety of patients admitted to coronary intensive care units and Cho et al. studied the effect of lavender on anxiety and vital signs of patients undergoing percutaneous coronary intervention (PCI) in intensive care units. The results of these studies showed that aromatherapy could reduce anxiety [11, 12]. Babatabar Darzi et al. examined the effects of rose and lavender on anxiety, pain at the incision site, and extubation time after open-heart surgery, and Akbari et al. studied the effect of peppermint essential oil on intravenous catheter pain and anxiety in ischemic heart patients. The results showed no significant difference in the level of anxiety among the study groups [13, 16]. Mashouf et al. studied the effect of lavender on agitation and hemodynamic parameters of the mechanically ventilated patients in the intensive care unit. They found the positive significant effect of lavender on agitated patients compared with the control group [8].

Apparently, patients admitted to ICUs experience varying degrees of anxiety and agitation. Therefore, concerning the importance and problems caused by anxiety and agitation in conscious patients admitted to intensive care units, it is necessary to use appropriate strategies and control these symptoms [3, 4, 6, 8, 13]. Since nurses have the most contact with patients, they must use different nonpharmacological interventions to control the anxiety and agitation of conscious patients admitted to the ICUs because such interventions are easy to access and inexpensive with lower side effects compared with pharmacological ones [10]. Therefore, this study was aimed at comparing the effects of lavender aromatherapy and Citrus aurantium aromatherapy on anxiety and agitation of the conscious patients admitted to ICUs in 2019.

## 2. Methods

### 2.1. Study Design and Setting

The parallel randomized placebo-controlled trial was performed on conscious patients admitted to the intensive care units of Afzalipour and Bahonar Hospitals in Kerman city in southeastern Iran. The research settings were Bahonar Hospital, the largest trauma center in southeastern Iran with 4 intensive care units and 12 active beds in each ward, and Afzalipour Hospital with three intensive care units (general ICU with 10 beds, surgical ICU with 8 beds, and poisoning ICU with 7 beds). Conscious patients admitted to these wards were the study samples.

### 2.2. Sample Size and Sampling

The following formula and previous studies were used to estimate the sample size in the present study [17].

(1) $$\begin{array}{c} n=\frac{\left(z_{\left(1-\left(\alpha /2\right)\right)}+z_{1-\beta }\right)2{\left(S_{1}^{2}+S_{2}^{2}\right)}^{2}}{{\left(\mu_{1}-\mu_{2}\right)}^{2}},\\ n^{\prime }=n*\sqrt{g-1}. \end{array}$$

The 95 percent confidence coefficient was calculated, so the confidence interval was 1.96. The type II error was 10% (1.63). In the study of Farshbaf-Khalili et al., the mean (±SD) scores of the anxiety in the lavender and the control groups were 34.2 ± 5.79 and 40.53 ± 8.98, respectively [17]. *n*′ is how to adjust the sample size based on the number of study groups. Therefore, the sample size was adjusted based on three study groups. Therefore, the number of samples required in each group was 50 individuals, who were randomly divided into three groups. Inclusion criteria were patients aged 18-60 years [18] with ability to read and write [19], those with normal olfactory sense [18], those with stable hemodynamic status [5], those who should not take sedatives and drugs 3 hours before or during the intervention [5], those with no asthma and other chronic respiratory problems, those with no intubation during the last 24 hours [15], those with no severe anxiety disorder diagnosed by a doctor, and those with no eczema and allergies to plants and citrus [19]. Exclusion criterion was the need to receive sedatives during the intervention.

**Figure 1:**
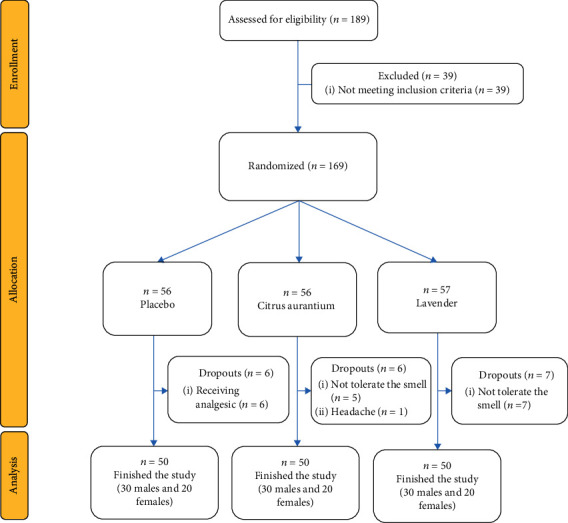
The flow diagram of the study.

Eligible patients were included in the study by convenience sampling method and were divided into two intervention groups (lavender and Citrus aurantium) and a placebo group by using a stratified block randomization method (stratum: gender, age (±2), and addiction). Labels A, B, or C (A: lavender, B: placebo, and C: Citrus aurantium) were assigned to the groups, and the block size was six. The randomization list was generated by using free online software (https://www.sealedenvelope.com/simple-randomiser/v1/lists). The fourth author generated the randomization list, and the first author enrolled the participants and assigned them to the three groups. Totally, 189 samples were assessed for eligibility, of which 169 eligible participants were allocated to the three groups. Finally, 50 participants finished the study (Figure 1).

### 2.3. Measurement

Demographic information form and two questionnaires were used to collect information. Demographic and background information includes age, sex, marital status, type of disease, level of education, living place, history of asthma or allergies, underlying diseases, vital signs, painkillers used for the patient, disease diagnosis, sedatives taken by the patient, history of admission to the intensive care unit, history of hospital stay, use or nonuse of sedatives and analgesics, dosage of sedatives and analgesics, and addiction.

The State-Trait Anxiety Inventory (STAI) was used to measure anxiety. The 20-item inventory consists of two parts of trait anxiety and state anxiety that assess the person's feelings at present moment and at the time of response. This study only used state anxiety subscale. Scoring was on a four-point scale from one (not at all) to four (very much so). The following guidelines are recommended for the interpretation of scores: 20-31, mild anxiety; 32-42, mild to moderate anxiety; 42-53, moderate to severe anxiety; 54-64, relatively severe anxiety; and ≥65, severe anxiety [20, 21]. The validity and reliability of the inventory were confirmed in a sample of Iranian adult students [20]. In the present study, the alpha Cronbach of state anxiety subscale was 0.85.

Sessler et al. validate the Richmond Agitation-Sedation Scale (RASS) in 2002. RASS is a 10-point scale, with four levels of anxiety or agitation (+1: restless, +2: agitated, +3: very agitated, and +4: combative), one level to denote a calm and alert state (0), and 5 levels of sedation (−1: drowsy, −2: light sedation, −3: moderate sedation, −4: deep sedation, and−5: unarousable). The RASS is a valid and reliable tool for measuring sedation-agitation in patients with/without mechanical ventilation and with/without sedatives [22, 23]. The validity and reliability of the scale were confirmed in a sample of Iranian intensive care patients [23]. In the present study, the Intraclass Correlation Coefficient (ICC) of RASS was 0. 86.

### 2.4. Intervention and Data Collection

Patients were divided into three groups of lavender aromatherapy, Citrus aurantium aromatherapy, and placebo by convenience sampling and randomized allocation method. Questionnaires were completed before, immediately, one hour, and three hours after the intervention. Note that in the study setting, painkillers and haloperidol were used in case of an anxious and agitated patient. In addition, the doctor might order psychiatric counseling for the patient.

In the placebo group, in addition to the routine care, placebo (normal saline) was used. First, the STAI and RASS were completed by interviewing. A 4 × 4 gauze saturated with 5 drops of normal saline was placed at a distance of 10 cm from the patient's nose (the gauze saturated with normal saline was attached to the patient's collar). The patient was asked to inhale it for 30 minutes ([24] #35). The questionnaires were recompleted by interviewing the patient immediately and one and three hours later.

In the group of lavender aromatherapy, in addition to the routine care, lavender essential oil was used. First, the questionnaires were completed by interviewing the patient. A 4 × 4 gauze saturated with 5 drops of lavender (manufactured by Tabib Daru Company, Mashhad, Iran) was placed at a distance of 10 cm from the patient's nose (the gauze saturated with lavender was attached to the patient's collar) ([24] #35). The patient was asked to inhale it for 30 minutes. The questionnaires were recompleted by interviewing immediately and one and three hours later.

In the group of Citrus aurantium aromatherapy, in addition to the routine care, Citrus aurantium essential oil was used. First, the STAI and RASS were completed by interviewing the patient. A 4 × 4 gauze saturated with 5 drops of citrusaurantium (manufactured by Tabib Daru Company, Mashhad, Iran) was placed at a distance of 10 cm from the patient's nose (the gauze saturated with Citrus aurantium was attached to the patient's collar) ([24] #35). The patient was asked to inhale it for 30 minutes. The questionnaires were recompleted by interviewing the patient immediately and one and three hours later. It should be noted that the sampling was performed on the second day of the patient's admission to the intensive care unit at 6-8 pm (to reduce workload and prepare the patient for sleep at night). The intervention was performed 3 hours later in case the patient received sedatives.

### 2.5. Data Analysis

SPSS25 was used for data analysis. Descriptive statistics (frequency, percentage, mean, and standard deviation) were used to describe the demographic characteristics of patients and information about the disease. Mean and standard deviation were used to describe anxiety score, and frequency and percentage were used to describe agitation. Chi-square, Fisher's exact, ANOVA, and Kruskal-Wallis tests were used to evaluate the homogeneity of the three groups in terms of underlying variables. According to the parametric conditions (Shapiro-Wilk test and equality of variances), repeated measures analysis of variance was used to compare the anxiety score before, immediately, one hour, and three hours after the intervention within and between groups. Friedman and chi-square tests were also used to compare within- and between-group agitation, respectively. A significance level of 0.05 was considered.

### 2.6. Ethical Consideration

The present study was performed following acquisition of the code of ethics (IR.KMU.REC.1398.179, Registered 14 November 2019, https://en.irct.ir/trial/40827) from Kerman University of Medical Sciences, the code of clinical trial from Iranian registry of clinical trial (IRCT20170116031972N9), and coordination with the heads of Afzalipour and Bahonar hospitals in Kerman. The purpose of this study was explained to the patients. Patients' informed written consents were taken, and it was explained that this intervention did not interfere with their treatments and they could withdraw from the study at any time. Patients in all three groups were provided with routine care, and any physical, psychological, or social harm was prevented.

## 3. Results

The mean ages of the samples in the lavender, Citrus aurantium, and placebo groups were 37.26 ± 12.72, 35.56 ± 11.41, and 35.70 ± 10.58, respectively (*P* = 0.72 and *F* = 0.33). No significant difference was found between the three groups in variables of hospital type, level of education, and living place. No significant difference was observed between the three groups in history of hospital stay, disease diagnosis, history of addiction, and underlying disease. No significant difference was found between the three groups in receiving sedatives and analgesics three hours before the intervention. Furthermore, no significant difference was observed between the three groups in medications, including anticonvulsants, antidotes, antihypertensive drugs, supplements, antibiotics, gastrointestinal medications, and corticosteroids (*P* > 0.05).

No significant difference was observed between the three groups in vital signs (systolic blood pressure, diastolic blood pressure, oxygen saturation, and pain) except for heart rate (*P* > 0.05).

Owing to the fact that the score of state anxiety in the placebo group was significantly higher than that of the Citrus aurantium group before the intervention, so the preintervention score was considered as a confounding variable in the repeated measures ANOVA and its effect was controlled. The results of repeated measures ANOVA showed that group-time interaction was significant. In addition, the group and time variables independently cause significant changes in the mean score of anxiety (Table 1). The mean anxiety of the lavender group was 54.88, 52.88, 54.70, and 50.26 before, immediately, one hour, and three hours after the intervention, respectively. Anxiety of the lavender group significantly reduced after the intervention. The mean anxiety of the Citrus aurantium group decreased from 53.92 before the intervention to 52.26 immediately after the intervention, 53.66 one hour after the intervention, and 50.10 three hours after the intervention. Anxiety of the Citrus aurantium group significantly reduced after the intervention. The mean anxiety of the placebo group decreased from 57.78 before the intervention to 57.72 immediately after the intervention, 57.14 one hour after the intervention, and 56.62 three hours after the intervention. The placebo group's anxiety did not reduce significantly (Tables 1 and 2). The mean scores of anxiety in the lavender and Citrus aurantium groups were significantly lower than those of the placebo group immediately after the intervention. No significant difference was found between the two groups of lavender and Citrus aurantium immediately after the intervention. There was no significant difference in the anxiety score between the three groups one hour after the intervention. The mean anxiety scores in the lavender and Citrus aurantium groups were significantly lower than those of the placebo group three hours after the intervention. No significant difference was observed between the two groups of lavender and Citrus aurantium three hours after the intervention (Table 3).

The prevalence of restlessness/agitation in the lavender group was 72, 72, 50, and 28 percent before the intervention, immediately after the intervention, one hour after the intervention, and three hours after the intervention, respectively. The prevalence of restlessness/agitation in the Citrus aurantium group was 86, 82, 64, and 36 percent before the intervention, immediately after the intervention, one hour after the intervention, and three hours after the intervention, respectively. The prevalence of restlessness/agitation in the placebo group was 66, 66, 42, and 40 percent before the intervention, immediately after the intervention, one hour after the intervention, and three hours after the intervention, respectively. Restlessness/agitation reduced significantly in all three groups. Although restlessness/agitation in lavender and Citrus aurantium groups reduced more than the placebo, no significant difference was found between the three groups (Table 4).

## 4. Discussion

Anxiety and agitation are the main problems of conscious patients admitted to the intensive care units, which should be eliminated to provide high-quality care. The results of the present study showed that the level of anxiety of lavender and Citrus aurantium groups reduced significantly compared with that of the placebo group. There was no significant difference between the two groups of lavender and Citrus aurantium.

Cho et al. [11], Ziyaeifard et al. [25], Trambert et al. [26], and Hasanpour et al. [27] showed that lavender aromatherapy could reduce anxiety [11, 25–27]. The results of two systematic review studies also showed that essential oils of lavender, rose, orange, sage, and tangerine had a positive effect on reducing anxiety about childbirth and dental anxiety [28, 29]. Inhalation of lavender might affect some parameters related to anxiety in the body and thus reduce anxiety. It is believed that lavender inhibits the activity of hypothalamus-hypophysis-adrenal by affecting the nervous system and thus reduces the secretion of cortisol and increases the secretion of serotonin [30]. In contrast, Babatabar Darzi et al. [13] studied the effects of rose aromatherapy and lavender aromatherapy on anxiety and pain at the incision site and Akbari et al. [16] examined the effect of peppermint essence on pain and anxiety caused by intravenous catheterization in cardiac patients. They showed no significant difference in anxiety between the groups participating in the study [13, 16]. One of the reasons for the different results is different characteristics of the study, especially the study population in the two studies that were opposed to the present study. In addition, in the mentioned studies, the anxiety was measured in a population with invasive interventions, so anxiety of such patients may be higher than that of those who are not under invasive procedures. Acute procedures are reported to be associated with a lot of fear, and patients may experience a lot of anxiety before, after, and during the procedures, including angiography and open-heart surgery [13].

Shirzadegan et al. [31] also evaluated the effect of Citrus aurantium aroma on anxiety and fatigue in patients with myocardial infarction. The results of this study showed that Citrus aurantium significantly reduced patients' anxiety. Aromas provide relaxation by stimulating the olfactory system [31]. Citrus aurantium essential oil stimulates the central nervous system, improves mood, and has sedative, antispasmodics, and diuretic effects.

The results related to the effect of lavender on agitation showed that the majority of samples in all three groups were agitated before the intervention, but agitation in all three groups decreased after the intervention. Although agitation in the lavender group reduced compared with that in the placebo group, no significant difference was found between the three groups. Watson et al. [32] examined the effect of lavender on agitated behaviors in older people and showed that lavender did not reduce agitation of the older people [32]. However, Mashouf et al. [8] in Iran showed that lavender aromatherapy had a positive effect on the agitation of mechanically ventilated patients in the intensive care unit compared with the control group [8]. The results of this study do not support the results of the present study. It should be noted that the effects of lavender essential oil are short. Zarifnejad et al. [33] believe that lavender has sedative but short effects [33]. In addition, the duration (one hour vs. half an hour), patients (mechanically ventilated vs. conscious patients), and the way the aroma is spread (using a humidifier device and nebulous vs. saturation and attachment of gauze to clothes) are other reasons for the difference between the results of the present study and the study of Mashouf et al. [8]. It should be noted that no study investigated the effect of Citrus aurantium on agitation. Therefore, further research is needed in this area. Researchers believe that plant aromas can activate olfactory nerve cells during aromatherapy, and nerve cells release different neurotransmitters, such as endorphin, noradrenaline, and serotonin, depending on the type of aroma, which can have rapid effects on reducing the level of anxiety and increasing comfort [34, 35]. Since agitation occurs because of patient's discomfort and anxiety [1] and according to the results of this study and previous studies on the effect of aromatherapy on anxiety, the effects of aromatherapy on agitation could be positive.

The present study had some limitations. Since patients admitted to intensive care units are not in a good condition and face many physical, mental, and psychological changes, the patients themselves were reluctant to participate in the study. Since severe pain and drug use before and during the study could be effective in assessing anxiety and agitation, not receiving sedatives and drugs three hours before or during the intervention was considered as an inclusion criterion. Therefore, a number of patients were excluded from the study because of pain and drug intake, which prolonged the sampling process.

## 5. Conclusions

The results of this study showed that lavender and Citrus aurantium essential oils reduced anxiety and agitation in conscious patients in the ICUs. Owing to the fact that one of the major challenges for intensive care units is the prevalence of anxiety and agitation, healthcare providers must correctly identify and control patients' anxiety and agitation. Healthcare providers have to use different ways to control them. Aromatherapy can affect one's mind, body, and soul and reduce anxiety by creating a sense of relaxation. It is not clear how aromatherapy can reduce anxiety [5]. Since aromatherapy is an inexpensive, easy, safe, and noninvasive method, it may be used as a beneficial intervention to reduce anxiety and agitation of conscious patients admitted to intensive care units. Further studies are needed to compare the efficacy of different essential oils to improve the state of anxiety and agitation management in the ICU setting.

## Figures and Tables

**Table 1:** Mean and standard deviation of the state anxiety of research units in three groups of lavender, Citrus aurantium, and placebo.

Group	Lavender		Citrus aurantium		Placebo	
Variable	Mean	SD	Mean	SD	Mean	SD
Before intervention	54.88	6.29	53.92	7.96	57.78	8.28
Immediately after intervention	52.88	4.74	52.26	6.55	57.72	8.20
One hour after the intervention	54.70	4.97	53.66	7.11	57.14	7.96
Three hours after the intervention	50.26	6.05	50.10	7.10	56.62	8.02

Source of change	Sum of squares		Degree of freedom	*F*	*P* value	Effect size
Group	681.98		2	9.04	<0.001	0.11
Time	558.43		1.79	20.51	<0.001	0.12
Group∗time	358.72		3.58	6.59	<0.001	0.08
Preintervention anxiety score	10487.58		1	277.94	<0.001	0.66
Error	5509.14		146			

**Table 2:** Results of Bonferroni test for comparing within-group variances in the mean anxiety at different times.

Group	Time (I)	Time (J)	Mean differences (I-J)	Standard error	*P* value
Lavender	Three hours after intervention	One hour after intervention	-4.68	0.85	<0.001
		Immediately after intervention	-2.85	0.70	<0.001
	One hour after intervention	Immediately after intervention	1.82	0.65	0.02

Citrus aurantium	Three hours after intervention	One hour after the intervention	-4.14	0.86	<0.001
		Immediately after intervention	-2.74	0.70	<0.001
	One hour after intervention	Immediately after intervention	1.41	0.65	0.10

Placebo	Three hours after intervention	One hour after intervention	0.30	0.86	>0.99
		Immediately after intervention	-0.29	0.71	>0.99
	One hour after intervention	Immediately after intervention	-0.59	0.66	>0.99

**Table 3:** Results of Bonferroni test for comparing between-group variances in the mean anxiety at different times.

Time	Group (I)	Group (J)	Mean differences (I-J)	Standard error	*P* value
Immediately after intervention	Lavender	Citrus aurantium	-0.11	0.66	>0.99
		Placebo	-2.62	0.67	<0.001
	Citrus aurantium	Placebo	-2.51	0.68	0.001

One hour after intervention	Lavender	Citrus aurantium	0.30	0.71	>0.99
		Placebo	-0.21	0.72	>0.99
	Citrus aurantium	Placebo	-0.52	0.73	>0.99

Three hours after intervention	Lavender	Citrus aurantium	-0.23	1.29	>0.99
		Placebo	-5.19	1.30	<0.001
	Citrus aurantium	Placebo	-4.96	1.32	0.001

**Table 4:** Frequency and percentage of agitation of research units in three groups of lavender, Citrus aurantium, and placebo.

Group		Lavender		Citrus aurantium		Placebo		Chi-square test	*P* value
Variable		Frequency	Percent	Frequency	Percent	Frequency	Percent		
Before intervention	Alert and calm	14	28	7	14	17	34	5.57	0.06
	Restless/agitated	36	72	43	86	33	66		

Immediately after intervention	Alert and calm	14	28	9	18	17	34	3.34	0.19
	Restless/agitated	36	72	41	82	33	66		

One hour after intervention	Alert and calm	25	50	18	36	29	58	4.97	0.08
	Restless/agitated	25	50	32	64	21	42		

Three hours after intervention	Alert and calm	36	72	32	64	30	60	1.65	0.44
	Restless/agitated	14	28	18	36	20	40		

Freidman test		46.98		55.57		33.00			
*P* value		<0.001		<0.001		<0.001			

## Data Availability

Data are available by contacting with the corresponding author by email.
